# Muscle and bone mass in middle‐aged women: role of menopausal status and physical activity

**DOI:** 10.1002/jcsm.12547

**Published:** 2020-02-03

**Authors:** Sarianna Sipilä, Timo Törmäkangas, Elina Sillanpää, Pauliina Aukee, Urho M. Kujala, Vuokko Kovanen, Eija K. Laakkonen

**Affiliations:** ^1^ Gerontology Research Center, Faculty of Sport and Health Sciences University of Jyväskylä Jyväskylä Finland; ^2^ Department of Obstetrics and Gynecology, Pelvic Floor Research and Therapy Unit Central Finland Central Hospital Jyväskylä Finland; ^3^ Faculty of Sport and Health Sciences University of Jyväskylä Jyväskylä Finland

**Keywords:** Sarcopenia, Osteoporosis, Midlife, Female, Sex hormones

## Abstract

**Background:**

Women experience drastic hormonal changes during midlife due to the menopausal transition. Menopausal hormonal changes are known to lead to bone loss and potentially also to loss of lean mass. The loss of muscle and bone tissue coincide due to the functional relationship and interaction between these tissues. If and how physical activity counteracts deterioration in muscle and bone during the menopausal transition remains partly unresolved. This study investigated differences between premenopausal, early perimenopausal, late perimenopausal, and postmenopausal women in appendicular lean mass (ALM), appendicular lean mass index (ALMI), femoral neck bone mineral density (BMD) and T score. Furthermore, we investigated the simultaneous associations of ALM and BMD with physical activity in the above‐mentioned menopausal groups.

**Methods:**

Data from the Estrogen Regulation of Muscle Apoptosis study were utilized. In total, 1393 women aged 47–55 years were assigned to premenopausal, early perimenopausal, late perimenopausal, and postmenopausal groups based on follicle‐stimulating hormone concentration and bleeding diaries. Of them, 897 were scanned for ALM and femoral neck BMD by dual‐energy X‐ray absorptiometry and ALMI (ALM/height^2^) and neck T scores calculated. Current level of leisure‐time physical activity was estimated by a validated self‐report questionnaire and categorized as sedentary, low, medium, and high.

**Results:**

Appendicular lean mass, appendicular lean mass index, femoral neck bone mineral density, and and T score showed a significant linear declining trend across all four menopausal groups. Compared with the postmenopausal women, the premenopausal women showed greater ALM (18.2, SD 2.2 vs. 17.8, SD 2.1, *P* < 0.001), ALMI (6.73, SD 0.64 vs. 6.52, SD 0.62, *P* < 0.001), neck BMD (0.969, SD 0.117 vs. 0.925, SD 0.108, *P* < 0.001), and T score (−0.093, SD 0.977 vs −0.459, SD 0.902, *P* < 0.001). After adjusting for potential confounding pathways, a higher level of physical activity was associated with greater ALM among the premenopausal [*β* = 0.171; confidence interval (CI) 95% 0.063–0.280], late perimenopausal (*β* = 0.289; CI 95% 0.174–0.403), and postmenopausal (*β*=0.278; CI 95% 0.179–0.376) women. The positive association between femoral neck BMD and level of physical activity was significant only among the late perimenopausal women (*β* = 0.227; CI 95% 0.097–0.356).

**Conclusions:**

Skeletal muscle and bone losses were associated with the menopausal transition. A higher level of physical activity during the different menopausal phases was beneficial, especially for skeletal muscle. Menopause‐related hormonal changes predispose women to sarcopenia and osteoporosis and further to mobility disability and fall‐related fractures in later life. New strategies are needed to promote physical activity among middle‐aged women. Longitudinal studies are needed to confirm these results.

## Introduction

Female sex hormones have wide‐ranging effects on women's health and well‐being over the lifespan. During midlife, women experience drastic hormonal changes due to ovarian aging and the consequent menopausal transition. The menopausal transition phase includes elevation of the serum follicle‐stimulating hormone (FSH) concentration and a decline in the estradiol concentration, both of which exhibit wide interindividual variation.^1^, ^2^, ^3^ Hormonal changes start approximately 5 years before and continue years after the final menstrual period.^1^, ^2^, ^4^ As the average menopausal age varies between 46 and 52 years,^5^ women spend more than one‐third of their lives in postmenopausal status. To understand and to promote women's health and well‐being during aging, the role of the menopausal transition on health determinants that go beyond the reproductive functions and organs need to be investigated and acknowledged.

Muscle and bone mass decline with aging, increasing the risk for sarcopenia and osteoporosis in later life. The contribution of menopause‐related hormonal changes, especially in oestrogens, is known to lead to bone loss through increased bone turnover with a net deficit in bone formation relative to bone resorption.^6^, ^7^ Earlier studies have shown a progressive decline in hip bone mineral density (BMD) from premenopausal to postmenopausal status and a significant association between BMD decline and either an increase in FSH^8^ or a decline in estradiol levels.^4^ A few studies also suggest that menopausal hormonal changes have an effect on the decline in lean mass (LM) among middle‐aged women^9^, ^10^ and that the decline continues up to 2 years after the final menstrual period.^9^ We have also shown a 3% net increase in total body LM and a 5% increase in thigh muscle cross‐sectional area after 1 year of hormone replacement therapy, started during the early postmenopausal years, compared with placebo treatment.^11^ Similarly, our genetically controlled case control study showed a 2% larger thigh muscle cross‐sectional area in sisters using hormone replacement therapy compared with their non‐using cotwins.^12^ These findings suggest that muscle tissue may be sensitive to menopausal hormonal changes in middle‐aged women.

Muscle and bone tissues have a close developmental and functional relationship.^13^, ^14^ Accumulating evidence shows that both muscle and bone have endocrine and paracrine properties and that muscle and bone cells secrete numerous biochemical compounds that interact with each other.^15^, ^16^, ^17^ According to the biomechanical coupling theory, bone adapts its mass, architecture, and strength to changes in stress and strain induced by gravitational loading and muscle activity.^18^, ^19^, ^20^ Accordingly, adaptations of bone and muscle tissue to reduced or increased loading conditions coincide.^13^, ^14^, ^21^, ^22^, ^23^ Because of the obvious beneficial effects of physical activity on muscle and bone health, physical activity is recommended for the prevention and treatment of osteoporosis^24^ and sarcopenia.^25^ However, hormonal changes seem to be the major contributors to the changes in muscle^11^, ^12^ and bone tissue^26^ in women undergoing the menopausal transition, whereas the level of physical activity seems to play a lesser role during this phase of life. Whether physical activity is an effective tool to counteract potential deterioration in muscle and bone during the menopausal transition and thus postpone or prevent sarcopenia and osteoporosis in later life remains partly unresolved.

This study investigated differences between 47 to 55‐year‐old premenopausal, early perimenopausal, late perimenopausal, and postmenopausal women in appendicular LM (ALM), appendicular LM index (ALMI), femoral neck BMD, and T‐score. We also investigated simultaneous associations of ALM and femoral neck BMD with current physical activity in the four menopausal groups.

## Materials and methods

### Study design and participants

Data from the Estrogenic Regulation of Muscle Apoptosis (ERMA) study were utilized. A detailed description of participant recruitment and categorization into menopausal groups has been reported earlier.^27^ In brief, women aged 47 to 55 years living in the city of Jyväskylä or neighbouring municipalities were randomly selected from the Finnish National Registry. Exclusion criteria were a self‐reported body mass index > 35 kg/m^2^, being currently pregnant or lactating, conditions affecting ovarian function, oral or transdermal oestrogen‐containing hormonal preparations or other medications affecting ovarian function, and chronic diseases or medications seriously affecting muscle function. A written invitation to take part in the study was sent to 6878 potential participants. The response rate was 47%. Eligible participants (*n* = 1627) were invited and 1393 came to the laboratory for a health interview and to give a fasting blood sample. Participants who had reported serious or unclear health conditions were examined by a physician to ensure safe participation in the measurements. All study participants provided a written informed consent. The study was approved by the ethics committee of the Central Finland Health Care District (K‐SSHP Dnro 8U/2014).

### Menopausal status

Participants' menopausal status was determined based on serum FSH concentration and menstrual cycle reported by monthly diaries and following the Stages of Reproductive Aging Workshop criteria.^1^ Hormone analyses were performed from fasting serum samples and in women with a menstrual cycle, during cycle days 1–5. Serum was separated by centrifugation for 10 min at 2200 ×*g*. FSH and 17β‐estradiol (E2) levels were immunoassayed using IMMULITE® 2000 XPi (Siemens Healthcare Diagnostics, UK). A participant was categorized as premenopausal if she had FSH < 9.5 IU/L or a regular menstrual cycle and FSH < 17 IU/L; early perimenopausal if she had FSH 17–25 IU/L or an irregular menstrual cycle and FSH 9.5–30 IU/L; late perimenopausal if she had FSH 25–30 IU/L or had experienced occasional menstrual bleeding during the past 3 months and FSH > 30 IU/L; and postmenopausal if she had experienced no menstrual bleeding during the past 6 months and FSH > 30 IU/L or no menstrual bleeding during the past 3 months and FSH > 39 IU/L or very high FSH (>130 IU/L) with or without occasional menstrual bleeding. For women with incomplete menstrual cycle information, the categorization was based solely on FSH level and stricter cutoff values were applied (Pre, FSH < 15 IU/L; EarlyPeri, FSH 15–25 IU/L; LatePeri, FSH 25–39 IU/L; and postmenopausal, FSH >39 IU/L). The numbers of participants in each category were premenopausal 235, early perimenopausal 180, late perimenopausal 193, and postmenopausal 289.

### Muscle mass and bone mineral density measurements

In total, 897 participants were scanned for femoral neck BMD (g/cm^2^) and ALM as a surrogate for muscle mass by dual‐energy X‐ray absorptiometry (DXA, LUNAR Prodigy, GE Healthcare) after overnight fasting. Participants were scanned in a supine position in the centre of the table using the default scanning mode for total body and proximal femoral region automatically selected by prodigy software (Lunar Prodigy Advance Encore v. 14.10.022). To calculate ALMI, ALM was divided by height squared (kg/m^2^).^28^ The numbers of participants with a femoral neck T score below −1 (cutoff for osteopenia^29^), ALM below 15.02 kg (cutoff for sarcopenia^30^), and ALMI below 5.45 kg/h^2^ (cutoff for sarcopenia^31^) were calculated. DXA‐measured femoral neck BMD^32^ and ALM,^30^ which describes functional LM, are ‘gold standard’ assessments for osteopenia/osteoporosis and sarcopenia, respectively.

### Physical activity assessment

Current level of leisure‐time physical activity (PA) was assessed by a questionnaire with a seven‐point scale ranging from household chores to competitive sports.^12^ A similar scale has been validated against accelerometer‐based physical activity and mobility variables.^33^ Specifically, the response categories were (1) no activity exceeding activities of daily living, (2) light walking or outdoor activity one to two times per week, (3) light walking or outdoor activity several times per week, (4) brisk physical activity causing some decree of sweating and breathlessness one to two times per week, (5) brisk physical activity causing some degree of sweating and breathlessness several times per week, (6) physical activity causing sweating and rather strong shortness of breath several times per week, and (7) competitive sports and related training. For the current analysis, the categories were combined and/or labelled as follows: 1 and 2 = sedentary behaviour; 3 and 4 = a low level PA; 5 = a medium level of PA; and 6–7 = a high level of PA.

### Background variables and confounders

*Level of education* was assessed by a single question and categorized as primary, secondary, bachelor's, master's, or doctoral level. Because of the small number of participants in the primary and doctoral levels, the primary and secondary levels were combined and the bachelor's, master's, and doctoral levels were also combined. *Body mass* was measured with a digital scale and *height* with a stadiometer. *Body mass index* was calculated as body mass divided by height squared (kg/m^2^). Total body fat mass (kg) was measured by DXA.

Participants self‐reported their musculoskeletal diseases or conditions, previous bone fractures, gynecologic history, and use of medications. Data related to the use of hormonal contraception and other gynecological hormonal treatments (progestogen preparation for contraception, progestogen products to treat gynecological bleeding disorders) were collected with standardized questions. Current users and those who had used oestrogen‐containing medication (given orally or transdermally) during the last 3 months were wholly excluded from the ERMA study. Smoking status was self‐reported with standardized questions and categorized as current, former, and never.

Hand grip force was measured with an adjustable dynamometer chair (Good Strength, Metitur, Palokka, Finland) from the dominant hand in a sitting position with elbow flexed at 90°. The participant was instructed to squeeze the handle as forcefully as possible for 2 to 3 s, and the peak value out of three to five maximal trials was taken as the result.

### Statistical analysis

Means and standard deviations for the continuous variables and frequency distributions with percentages for the categorical variables were calculated. Differences between the groups in the categorical variables were assessed by cross tabulation and χ^2^ test. To analyse linear trends in the continuous background variables and in muscle and bone mass over the menopausal groups, the Welch test [a modification of one‐way analysis of variance (ANOVA) accommodating unequal group variances] was used. Adjustment for age was performed using univariate analysis of covariance. Tamhane's post hoc test was used to localize differences in the background variables between the menopausal groups. To explore indications for the timing of bone and muscle loss during the menopausal transition, the ALM, ALMI, BMD, and neck T score of the premenopausal women (reference) were compared with the corresponding values for the other three study groups using Dunnett's post hoc testing procedure.

**Figure 1:**
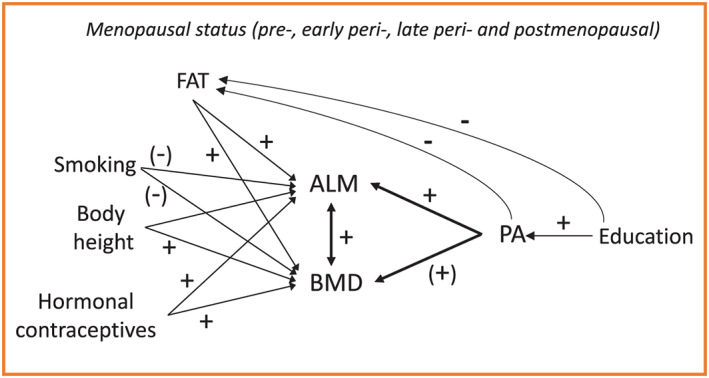
Theoretical model showing the four‐group multivariate linear model with main outcomes and confounders. + denotes to positive and − to negative theoretical and our empirical associations between the variables. (−) and (+) denote to theoretical associations between the variables. ALM, appendicular lean mass; BMD, bone mineral density; PA, physical activity.

To investigate the simultaneous association of ALM and BMD with physical activity, a four‐group multivariate linear model was constructed. In addition, a prespecified contrast was used to explore the differences between the premenopausal and early perimenopausal women combined and the late perimenopausal and postmenopausal women combined. Theoretically, meaningful and available confounders, which were significantly associated with the predictor or the outcome, were included in the models shown in *Figure* 1. Education was significantly associated with PA (χ^2^, *P* = 0.002); fat mass differed significantly between the physical activity (one‐way ANOVA, *P* < 0.001) and education (one‐way ANOVA, *P* = 0.03) groups; fat mass was associated with ALM (Pearson *r* = 0.229, *P* < 0.001) and BMD (Pearson *r* = 0.217, *P* < 0.001); body height was associated with ALM (Pearson *r* = 0.601, *P* < 0.001) and BMD (Pearson *r* =0.202, *P* < 0.001); and a significant increasing linear trend was observed in the ALM and the BMD from the non‐users of the contraceptives to the former users and to the current users (one‐way ANOVA, *P* < 0.001).

Although smoking status showed no statistically significantly association with either ALM or BMD, we decided to retain it in the model. Smoking is a known risk factor for osteoporosis, and it is suggested that smoking is inversely associated with peak torque and fat infiltration into muscle tissue among postmenopausal women.^34^, ^35^, ^36^ Significance level was set at 0.05.

## Results

### Baseline characteristics

As expected, the premenopausal women were the youngest and the postmenopausal women the oldest (*Table* 1). No significant age difference was observed between the premenopausal and early perimenopausal women. All other age‐related pairwise comparisons were, however, statistically significant (*P* < 0.001). Thus, the serum E2 concentration was the highest and FSH concentration lowest among the premenopausal women followed, in descending order, by the early perimenopausal, late perimenopausal, and postmenopausal women (*P* < 0.001 for all pairwise comparisons). No significant between‐group differences were observed in anthropometrics, total body fat mass, smoking status, musculoskeletal problems, previous bone fracture, or level of physical activity. As expected, the distribution of the use of hormonal contraception differed significantly between the groups; nearly half of the premenopausal women were current users, whereas among the perimenopausal and postmenopausal groups, the corresponding percentage varied from 26% to 32%. Because use of oestrogen containing medication was the exclusion criteria of the current study, the current users of hormonal contraception include only users of intrauterine device (95%) or progestin‐only pills (5%). Grip force was significantly lower in the postmenopausal than premenopausal and early perimenopausal women (*P* < 0.001 for both comparisons).

**Table 1:** Characteristics of the participants according to the four menopausal status groups

Participant characteristics	Pre *n* = 235	EarlyPeri *n* = 180	LatePeri *n* = 193	Post *n* = 289	*P* for trend
Age, year	50.6 (1.6)	50.7 (1.8)	51.7 (1.9)	52.6 (1.9)	<0.001
E2 (nmol/L)	0.627 (0.667)	0.403 (0.318)	0.262 (0.186)	0.144 (0.100)	<0.001
FSH (UI/L)	7.9 (3.5)	16.8 (4.8)	45.1 (20.1)	82.4 (28.4)	<0.001
Weight, kg	70.0 (10.0)	70.5 (11.3)	70.7 (11.0)	68.5 (11.1)	0.13
Height, cm	166 (5.3)	165 (5.5)	165 (6.0)	165 (6.0)	0.23
BMI	25 (3)	26 (4)	26 (4)	25 (4)	0.11
Fat mass, kg	24.4 (8.0)	25.3 (8.7)	26.3 (8.6)	24.4 (8.5)	0.06
Grip force, *N*	323.4 (60.6)	323.4 (61.3)	312.3 (61.2)	298.9 (54.2)	<0.001
Education, *n* (%)					0.11^a^
Primary	1 (0.4)	3 (1.7)	5 (3)	8 (3)	
Secondary	128 (55)	99 (55)	96 (50)	179 (62)	
Bachelor	27 (12)	24 (13)	26 (14)	41 (14)	
Master	64 (27)	43 (24)	54 (28)	49 (17)	
Doctoral	14 (6)	10 (6)	11 (6)	11 (4)	
Smoking, *n* (%)					0.83^a^
Never	156 (67)	122 (68)	123 (64)	194 (67)	
Former	63 (27)	45 (25)	49 (26)	73 (25)	
Current	14 (6)	12 (7)	19 (10)	21 (7)	
Hormonal contraception, *n* (%)					<0.001^a^
Never	90 (39)	94 (53)	110 (57)	160 (56)	
Former	36 (15)	27 (15)	28 (15)	54 (19)	
Current^b^	108 (46)	58 (32)	54 (28)	74 (26)	
Musculoskeletal problem, *n* (%)	78 (33)	63 (35)	77 (40)	99 (34)	0.49^a^
Previous bone fracture, *n* (%)	37 (16)	25 (14)	34 (18)	43 (15)	0.77^a^
Physical activity, *n* (%)					0.51^a^
Sedentary	21 (9)	24 (13)	16 (8)	35 (12)	
Low	68 (29)	50 (28)	54 (28)	78 (27)	
Moderate	90 (39)	70 (39)	90 (47)	118 (41)	
High	55 (24)	35 (20)	32 (17)	57 (20)	

Mean and (standard deviation) for the continuous variables and number of participants (*n*) and percentages (%) for the categorical variables.

BMI, body mass index; E2, 17β‐estradiol; FSH, follicle‐stimulating hormone.

a χ^2^ test.

b 95% used intrauterine device and 5% minipills.

### Muscle mass and bone mineral density in the four menopausal groups

Appendicular LM and ALMI showed a significant linear trend across the four menopausal groups (*Table* 2). ALM and ALMI were significantly higher in the premenopausal than postmenopausal women (*P* < 0.001 for both comparisons). The difference between premenopausal and early perimenopausal women in ALM (*P* = 0.27) and ALMI (*P* = 0.66) and between premenopausal and late perimenopausal women in ALMI (*P* = 0.09) were not statistically significant. The ALM was significantly higher among premenopausal compared with late perimenopausal women (*P* = 0.03). The relative proportion of participants with ALM below the sarcopenia cutoff value of 15.02 was 4–5% in the premenopausal and early perimenopausal groups and 7–8% in the late perimenopausal and postmenopausal groups. Accordingly, 2–3% of premenopausal and early perimenopausal women had ALMI below the sarcopenia cutoff value of 5.45 while the corresponding percentages in the late perimenopausal and postmenopausal women were 3–4%.

**Table 2:** Muscle and bone characteristics according to the four menopausal status groups

Muscle and bone characteristics	Pre *n* = 235	EarlyPeri *n* = 180	LatePeri *n* = 193	Post *n* = 289	*P*	*P* a
ALM, kg	18.6 (2.2)	18.3 (2.3)	18.1 (2.3)	17.8 (2.1)	<0.001	0.002
ALM < 15.02, *n* (%)	11 (5)	8 (4)	14 (7)	24 (8)	0.23^b^	0.39^b^
ALMI, kg/h^2^	6.73 (0.64)	6.68 (0.67)	6.60 (0.64)	6.52 (0.62)	<0.001	<0.001
ALMI < 5.45, *n* (%)	6 (3)	4 (2)	6 (3)	12 (4)	0.64^b^	0.68^b^
BMD, g/cm^2^	0.969 (0.117)	0.984 (0.123)	0.969 (0.130)	0.925 (0.108)	<0.001	0.003
Neck T score	−0.093 (0.977)	0.059 (0.977)	−0.091 (1.080)	−0.459 (0.902)	<0.001	0.002
T score < −1, *n* (%)	40 (17)	23 (13)	44 (24)	79 (27)	0.001^b^	0.04^b^

ALM, appendicular lean mass; ALMI, appendicular lean mass index; BMD, bone mineral density.

a Age‐adjusted.

b χ^2^ test.

BMD and T score also showed a significant trend over the four menopausal groups (*Table* 2). Neck BMD and T score were significantly higher in the premenopausal than postmenopausal women (*P* < 0.001 for both comparisons). The differences between premenopausal and early menopausal women in BMD (*P* = 0.42) and in T score (*P* = 0.28) were not statistically significant. Similarly, the difference between premenopausal and late perimenopausal women in BMD and in T score (*P* = 1.00 for both comparisons) were not statistically significant. The relative proportion of participants with a T score below −1 (cutoff for osteopenia) was 17% in the premenopausal and 13% in the early perimenopausal women and 24% in the late perimenopausal and 27% in the postmenopausal women.

### Associations between physical activity, muscle mass, and bone mineral density in the four menopausal groups

**Figure 2:**
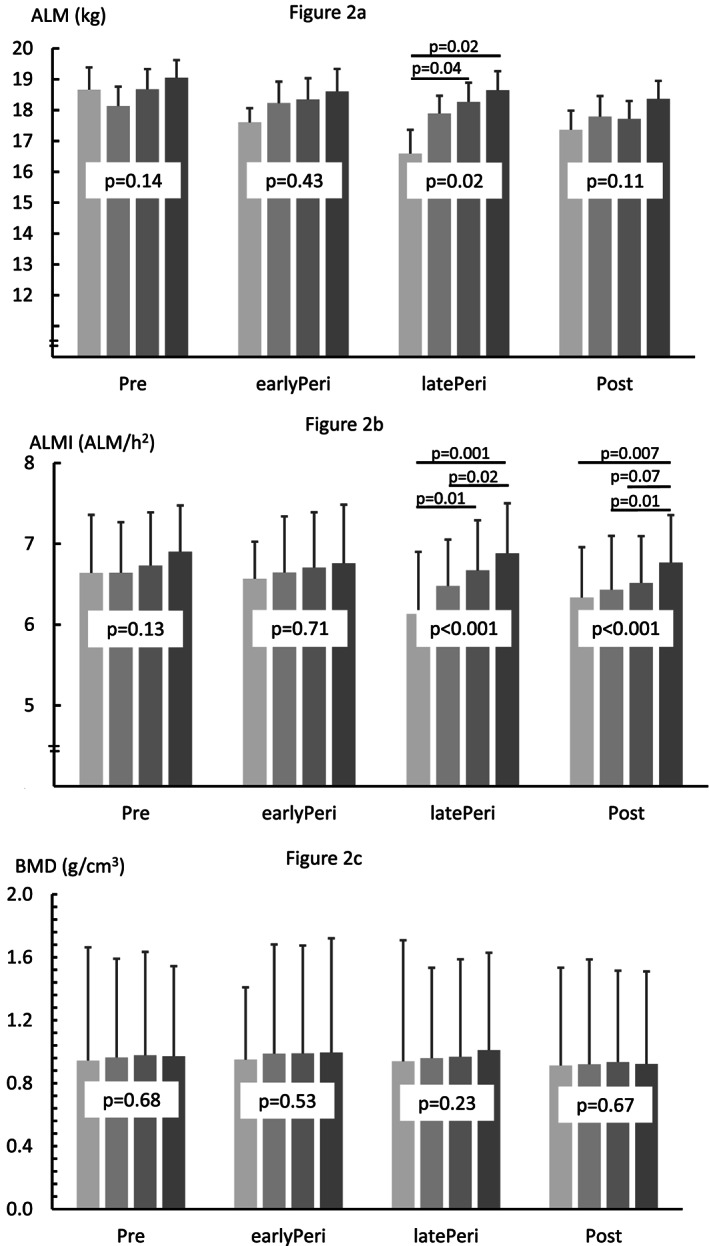
Means and standard deviations for appendicular lean mass (ALM), appendicular lean mass index (ALMI), and femoral neck bone mineral density (BMD) in physical activity categories by menopausal group. Bar colours from light to dark grey represent sedentary, low, medium, and high level of physical activity.

The differences in ALM, ALMI, and neck BMD between the physical activity groups are shown in *Figure* 2. ALM and ALMI were greater in the late perimenopausal women with a moderate or high level of physical activity than in their sedentary counterparts. ALMI was also greater in the late perimenopausal women with high physical activity than those with light physical activities. In postmenopausal group, the women with a high level of physical activity had greater ALMI than counterparts who were sedentary or women engaged with light physical activities. No significant differences between the physical activity groups were observed in neck BMD.

**Figure 3:**
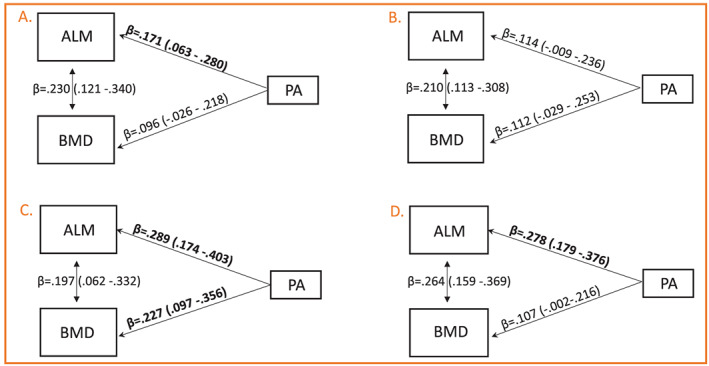
Four‐group multivariate linear model exploring the simultaneous association of appendicular lean mass (ALM) and femoral neck bone mineral density (BMD) with physical activity (PA). (A) Premenopausal, (B) early perimenopausal, (C) late perimenopausal, and (D) postmenopausal groups. The model has been adjusted for education, fat mass, body height, smoking, and use of hormonal contraceptives.

*Figure* 3 and *Table* S1 show the results for the simultaneous association of ALM and BMD with physical activity in the four menopausal groups adjusted for education, smoking, fat mass, body height, and use of hormonal contraceptives as presented in *Figure* 1. In the adjusted models, the associations between ALM and BMD varied between 0.197–0.264. In premenopausal, late perimenopausal, and postmenopausal women, a higher level of physical activity was associated with greater ALM while the association in the early perimenopausal women was borderline significant. These path coefficients were significantly higher among the late perimenopausal and postmenopausal than premenopausal and early postmenopausal groups (*P* = 0.02).

In the adjusted models that included ALM, BMD, and the above‐mentioned covariates, a higher level of physical activity was significantly associated with greater femoral neck BMD only in the late perimenopausal women; in the postmenopausal women, the association was borderline significant. No significant differences were observed in the path coefficients between the premenopausal and early menopausal groups and the late perimenopausal and postmenopausal groups (*P* = 0.30). All the path coefficients of the adjusted models are shown in the Supporting Information, *Table* S1.

## Discussion

This cross‐sectional study showed a linear declining trend in muscle mass and bone density across the menopausal groups and significantly lower values in muscle mass and bone density in the postmenopausal compared with premenopausal women. Moreover, we showed that a high level of physical activity, especially during and after the late perimenopausal phase, was associated with greater muscle mass. However, owing to the methods used in this study, the association between physical activity and BMD was less evident. In sum, these results indicate that muscle and bone tissues are sensitive to the menopausal transition and that physical activity is beneficial for muscle mass among middle‐aged women undergoing the menopausal transition. The influence of physical activity on BMD is, however, less clear and to determine this may require a longer postmenopausal follow‐up.

### Muscle mass and bone mineral density in the four menopausal groups

Although the deterioration in bone density due to menopause‐related hormonal changes has been recognized for decades,^7^ studies on the role of the menopausal transition on muscle mass remain scarce. In support of our findings on ALM and ALMI, a recent follow‐up study by Greendale et al.^9^ showed a significant annual decline of 0.06 kg (0.2%) in total body LM during 4 to 5 years of the menopausal transition. Moreover, in our previous intervention study among slightly older (50 to 57‐year‐old) postmenopausal women, we observed a 0.6% annual decline in total body fat‐free mass (FFM) among women who did not participate in the intervention.^11^ A similar trend in FFM has also been reported.^37^ LM and bioimpedance‐measured FFM are less accurate surrogates for muscle mass than ALM, as LM includes organ mass and FFM includes bone mass. Using ALM, the currently recommended method of measuring indications for sarcopenia, we found an average difference of 0.8 kg (4%) between the premenopausal and postmenopausal women. The significant difference in ALM and ALMI between the study groups and the slightly greater amount of participants below the sarcopenia cutoff points in late perimenopausal and early postmenopausal compared with premenopausal and early perimenopausal women indicate that menopause‐related hormonal changes may trigger the cascade that in some women eventually leads to sarcopenia. This is an important finding, as the decline in muscle mass continues and may even accelerate by aging as pointed out by Health ABC study.^38^ They showed a nearly 1% annual decline in leg LM among women between the ages of 70 and 79.

The biological mechanisms behind the loss of skeletal muscle mass due to menopause‐related hormonal changes remain partly unclear. The potential mechanisms may lie along the whole neuromuscular pathway, as the brain,^39^ motoneurons,^40^ skeletal muscle cells,^41^, ^42^ and tenoblasts and fibroblasts^43^ all express oestrogen receptors and thus are targets for these hormones. The most plausible structure through which oestrogen deprivation may have an effect on muscle mass is the muscle tissue itself.^44^ A recent review by Collins et al.^45^ suggests that cellular apoptosis pathways, including heat‐shock proteins, ligands for cell death receptors, and microRNAs that are known to target key players of the apoptotic pathways, are the strongest candidates for muscle mass decline during oestrogen deficiency. Moreover, muscle fibre atrophy may be due to the mitochondrial dysfunction that induces apoptosis and dysregulation of energetic pathways.^45^

Decline in muscle mass is one of the mechanisms underlying the aging‐related deterioration in muscle strength. However, the age‐induced decline in muscle strength may be up to three times greater than the decline in muscle mass,^38^ suggesting that neural and muscle quality‐related factors have a notable effect on muscle weakness in old age. Our earlier analysis among ERMA participants showed an average of 5% lower knee extension and 8% lower grip force in the postmenopausal compared with premenopausal women.^46^ The mean difference in ALM of 4% observed between the premenopausal and postmenopausal groups in the present study prompts the speculation that the menopause‐related decline in muscle mass is one of the mechanisms initiated already during midlife that underlie the decline in muscle force observed in women.

In this study, a significant difference in femoral neck BMD compared with the premenopausal group was evident only after the late perimenopause; the difference between premenopausal and postmenopausal women being 5%. However, nearly one‐fourth of the late perimenopausal participants had a T score below −1, suggesting that clinically meaningful changes in bone density occur as early as already during the perimenopause. This is supported by the Women's Health Across the Nation study,^47^ which showed an accelerated decline in femoral neck BMD during the perimenopausal years, with the greatest annual change occurring 1 to 2 years after the final menstrual period. The same study also suggested that the loss of BMD may be triggered by the elevation of serum FSH levels that occur during the late premenopausal years.^8^, ^48^

Adult bone is under constant remodeling via osteoblast‐driven bone formation and osteoclast‐induced bone resorption. Oestrogen deficiency results in increased bone turnover with a net deficit in bone formation relative to resorption and thus bone loss.^49^, ^50^ Trabecular bone, which is also represented in the femoral neck, seems to be especially sensitive to menopause‐related hormonal changes.^8^ The biological mechanisms behind oestrogen deprivation‐induced bone deterioration have been investigated for a long time. The results of these investigations have led to the development of effective pharmacologic therapies. Oestrogen‐containing hormone replacement therapies, which were among the first of these,^51^ have been followed by a number of other medications such as selective oestrogen receptor modulators.^52^ Due to their effectiveness on bone health and in fracture prevention,^53^ pharmacologic therapies are the first‐line treatments for low bone density among postmenopausal women. Although the importance of physical activity has also been acknowledged in the current recommendations, a detailed ‘prescription’ regarding its intensity, quality, and duration continues to be lacking.^54^

### Associations between physical activity, muscle mass, and bone mineral density in the four menopausal groups

In this study, the late perimenopausal and postmenopausal women who reported a higher level of physical activity had greater ALM and ALMI than their less physically active counterparts. Similar associations were not observed in the premenopausal or early perimenopausal groups. This may indicate that being physically active is especially important for women who already have a notable decline in sex hormone production, whereas among heathy premenopausal and early perimenopausal women, the hormonal milieu maintains muscle mass to a level that is adequate for negotiating the activities of daily life. This is supported by the fact that the sedentary premenopausal women had comparable or greater ALMI than the moderately physically active late perimenopausal or postmenopausal women.

The association between physical activity and femoral neck BMD was less evident than that between physical activity and ALM/ALMI. In the adjusted models, the association was significant in the late perimenopausal women and borderline significant in the postmenopausal women, and both associations were weaker than those observed between physical activity and ALM/ALMI. It may be that hormonal factors outweigh the potential effects of physical activity on femoral neck BMD during the menopausal transition, as the bone tissue is under strong hormonal regulation. It may also be that our participants did not engage in specific bone‐loading activities. Intervention studies show that regular physical activity several times a week is effective in increasing femoral neck BMD in women during their postmenopausal years.^19^, ^55^ However, to induce osteogenic effects on the femoral neck, intensive physical exercise such as brisk walking, running and jumping,^56^, ^57^, ^58^ or high‐load resistance training targeting the hip region^59^, ^60^ is required. For example, habitual walking at 4 km/h is not associated with improved hip BMD.^61^

Physical activity is known to be an important health‐enhancing lifestyle factor for everyone throughout the life course. However, several factors indicate that, in women, midlife is one of the life stages in which physical activity has particular importance. The hormonal changes that occur during midlife have effects not only on the musculoskeletal system but also on fat mass and its redistribution, leading in turn to increased risk for many chronic diseases.^62^ Experimental animal and human studies have indicated that the mechanisms underlying the changes in postmenopausal body composition are related to the accompanying decline in resting and total energy expenditure^63^ and to a reduced level of physical activity.^64^ Thus, more research is needed to identify effective physical activity strategies that are feasible and target the critical factors known to be influenced by menopause.

### Strengths and limitations

The main limitation of this study is the cross‐sectional design with group comparisons and correlative analysis, which do not enable causal interpretations. We may also have failed to take into account all of the important confounding factors, for example, individual genetic factors that may have an effect on muscle mass and bone density. Our earlier studies among postmenopausal women show that 60% of the variance in lower limb bone strength^65^ and 31% of that in knee extension strength^66^ are explained by genetic factors and that the association between muscle mass and bone strength derives from both genetic and environmental factors.^67^ In this study, the level of physical activity was self‐reported using a questionnaire, which mostly captures leisure‐time physical activity, leaving work‐related physical activity unnoticed. Typically, however, leisure‐time physical activity has shown stronger associations with better health metrics,^68^, ^69^ whereas physically heavy work may be associated with adversities.^70^ Self‐reports may also include bias by underestimating the number of low and overestimating the number of highly physically active participants.^71^ There is no reason, however, to assume that potentially imprecise reporting would differ across our menopausal status groups. This study also has several strengths. First, we utilized current recommendations and gold standards to evaluate muscle mass and BMD along with prevalence of sarcopenia and osteopenia. Second, our large population‐based sample of 47 to 55‐year‐old women was carefully categorized into four menopausal groups according to current international guidelines, utilizing menstrual diary records and hormone analysis. Third, our database, including comprehensive information on outcomes and potential confounding factors, offers reliable information that can be used in investigating the complex relationships between hormonal aging and muscle and bone health.

## Conclusions

This large‐scale cross‐sectional study showed a significant decline in muscle mass and bone density across the menopausal groups, suggesting that skeletal muscle and bone are sensitive to the menopausal transition and related hormonal changes. As physical activity was associated with greater muscle mass among the premenopausal, late perimenopausal, and postmenopausal women, engaging in a physically active lifestyle during midlife may reduce the risk for sarcopenia in later life. New strategies are needed to promote physical activity among adult women. Carefully designed longitudinal studies, which go beyond the menopausal transition and include measures that are sensitive to changes in muscle and bone and to the level of physical activity, are needed to confirm these results. Long‐term follow‐up studies are also needed to investigate whether menopause‐related changes in muscle and bone are associated with sarcopenia and osteoporosis in later life.

## Conflicts of interest

None declared.

## Supporting information


**Table S1.** Standardized estimates, standard errors and p‐values for path coefficients, covariance and residual variance terms drawn from a four–group multivariate linear model and presented in the premenopausal (PRE), early perimenopausal (ePERI), late perimenopausal (lPERI) and postmenopausal (POST) groups.
