# Supplementary material for: Muscle and bone mass in middle‐aged women: role of menopausal status and physical activity
Source: J Cachexia Sarcopenia Muscle. 2020 Feb 3;11(3):698–709. doi: 10.1002/jcsm.12547 (PMC7296268; doi:10.1002/jcsm.12547)
Supplement: Supplementary file 1 — Table S1. Standardized estimates, standard errors and p‐values for path coefficients, covariance and residual variance terms drawn from a four–group multivariate linear model and presented in the premenopausal (PRE), early perimenopausal (ePERI), late perimenopausal (lPERI) and postmenopausal (POST) groups. [file JCSM-11-698-s001.docx]

**Table S1. Standardized estimates, standard errors and p-values for path coefficients, covariance and residual variance terms drawn from a four–group multivariate linear model and presented in the premenopausal (PRE), early perimenopausal (ePERI), late perimenopausal (lPERI) and postmenopausal (POST) groups.**

|  | **PRE** | | |  | **ePERI** | | |  | **lPERI** | | |  | **POST** | | |
| --- | --- | --- | --- | --- | --- | --- | --- | --- | --- | --- | --- | --- | --- | --- | --- |
|  | **Est.** | **SE** | **p-value** |  | **Est.** | **SE** | **p-value** |  | **Est.** | **SE** | **p-value** |  | **Est.** | **SE** | **p-value** |
| Associations with ALM |  |  |  |  |  |  |  |  |  |  |  |  |  |  |  |
| PA | **0.17** | **0.06** | **0.002** |  | 0.11 | 0.06 | 0.07 |  | **0.29** | **0.06** | **<0.001** |  | **0.28** | **0.05** | **<0.001** |
| Total FM | **0.12** | **0.05** | **0.01** |  | **0.22** | **0.05** | **<0.001** |  | **0.18** | **0.05** | **<0.001** |  | **0.29** | **0.05** | **<0.001** |
| Former smoker vs. non- smoker | 0.09 | 0.05 | 0.09 |  | 0.06 | 0.07 | 0.35 |  | 0.09 | 0.0 | 0.11 |  | 0.01 | 0.05 | 0.80 |
| Current smoker vs. non-smoker | 0.02 | 0.08 | 0.75 |  | 0.03 | 0.07 | 0.64 |  | -0.03 | 0.07 | 0.68 |  | 0.03 | 0.05 | 0.57 |
| Body height | **0.58** | **0.05** | **<0.001** |  | **0.61** | **0.05** | **<0.001** |  | **0.63** | **0.05** | **<0.001** |  | **0.55** | **0.04** | **<0.001** |
| Former HC user vs. non- HC user | 0.00 | 0.06 | 0.94 |  | -0.05 | 0.07 | 0.46 |  | 0.00 | 0.06 | 0.97 |  | 0.10 | 0.06 | 0.08 |
| HC User vs. non-user | 0.03 | 0.06 | 0.65 |  | 0.06 | 0.06 | 0.32 |  | 0.10 | 0.06 | 0.10 |  | 0.04 | 0.05 | 0.49 |
| Mid education vs. Low education | **-0.11** | **0.05** | **0.04** |  | 0.03 | 0.06 | 0.61 |  | -0.07 | 0.05 | 0.20 |  | -0.02 | 0.05 | 0.64 |
| High education vs. Low education | -0.08 | 0.06 | 0.17 |  | 0.05 | 0.07 | 0.42 |  | -0.05 | 0.06 | 0.40 |  | -0.01 | 0.04 | 0.86 |
|  |  |  |  |  |  |  |  |  |  |  |  |  |  |  |  |
| Associations with BMD  PA | 0.10 | 0.06 | 0.12 |  | 0.11 | 0.07 | 0.12 |  | **0.23** | **0.07** | **0.001** |  | 0.11 | 0.06 | 0.05 |
| Total FM | 0.11 | 0.07 | 0.13 |  | **0.34** | **0.07** | **<0.001** |  | **0.24** | **0.07** | **<0.001** |  | **0.27** | **0.06** | **<0.001** |
| Former smoker vs. non- smoker | -0.01 | 0.07 | 0.91 |  | 0.03 | 0.08 | 0.75 |  | -0.05 | 0.08 | 0.51 |  | 0.01 | 0.06 | 0.82 |
| Current smoker vs. non-smoker | -0.10 | 0.06 | 0.13 |  | -0.09 | 0.09 | 0.33 |  | -0.07 | 0.07 | 0.34 |  | 0.05 | 0.05 | 0.37 |
| Body height | **0.31** | **0.06** | **<0.001** |  | **0.19** | **0.08** | **0.02** |  | **0.19** | **0.08** | **0.01** |  | 0.09 | 0.06 | 0.14 |
| Former HC user vs. non-user | -0.02 | 0.07 | 0.80 |  | 0.02 | 0.09 | 0.80 |  | -0.02 | 0.07 | 0.83 |  | 0.04 | 0.07 | 0.55 |
| HC user vs. non-user | -0.01 | 0.07 | 0.95 |  | **0.20** | **0.08** | **0.02** |  | **0.18** | **0.07** | **0.02** |  | **0.20** | **0.06** | **0.001** |
| Mid education vs. Low education | -0.08 | 0.07 | 0.26 |  | 0.02 | 0.07 | 0.83 |  | -0.04 | 0.07 | 0.58 |  | 0.09 | 0.06 | 0.16 |
| High education vs. Low education | -0.02 | 0.07 | 0.79 |  | 0.09 | 0.08 | 0.23 |  | -0.07 | 0.07 | 0.34 |  | 0.08 | 0.06 | 0.17 |
|  |  |  |  |  |  |  |  |  |  |  |  |  |  |  |  |
| Mid education vs. Low education | 0.08 | 0.07 | 0.24 |  | 0.09 | 0.11 | 0.41 |  | -0.04 | 0.09 | 0.64 |  | **0.13** | **0.06** | **0.03** |
| High education vs. Low education | **0.19** | **0.07** | **0.01** |  | 0.14 | 0.08 | 0.10 |  | -0.01 | 0.08 | 0.95 |  | 0.06 | 0.07 | 0.37 |
|  |  |  |  |  |  |  |  |  |  |  |  |  |  |  |  |
| Associations with FM  PA | **-0.31** | **0.06** | **<0.001** |  | **-0.28** | **0.07** | **<0.001** |  | **-0.36** | **0.07** | **<0.001** |  | **-0.32** | **0.05** | **<0.001** |
| Mid education vs. Low education | -0.05 | 0.07 | 0.50 |  | 0.08 | 0.07 | 0.25 |  | -0.03 | 0.09 | 0.73 |  | 0.09 | 0.06 | 0.13 |
| Mid education vs. Low education | -0.07 | 0.07 | 0.35 |  | -0.09 | 0.09 | 0.27 |  | -0.10 | 0.07 | 0.20 |  | -0.07 | 0.06 | 0.28 |
|  |  |  |  |  |  |  |  |  |  |  |  |  |  |  |  |
| **Covariation** |  |  |  |  |  |  |  |  |  |  |  |  |  |  |  |
| ALM and BMD | **0.23** | **0.06** | **<0.001** |  | **0.21** | **0.05** | **<0.001** |  | **0.20** | **0.07** | **0.004** |  | **0.26** | **0.05** | **<0.001** |
|  |  |  |  |  |  |  |  |  |  |  |  |  |  |  |  |
| **Residual variance** |  |  |  |  |  |  |  |  |  |  |  |  |  |  |  |
| Total FM | **0.90** | **0.04** | **<0.001** |  | **0.90** | **0.05** | **<0.001** |  | **0.86** | **0.05** | **<0.001** |  | **0.89** | **0.04** | **<0.001** |
| ALM | **0.60** | **0.06** | **<0.001** |  | **0.56** | **0.06** | **<0.001** |  | **0.49** | **0.05** | **<0.001** |  | **0.56** | **0.05** | **<0.001** |
| BMD | **0.88** | **0.04** | **<0.001** |  | **0.80** | **0.06** | **<0.001** |  | **0.85** | **0.05** | **<0.001** |  | **0.87** | **0.04** | **<0.001** |

PA=Physical activity; ALM=Appendicular lean mass; BMD=Bone mineral density; FM=Fat mass; HC=Hormone contraceptive

Note. Bold typeface indicates statistically significant coefficient.
